# In silico modeling of phosphorylation dependent and independent c-Myc degradation

**DOI:** 10.1186/s12859-019-2846-x

**Published:** 2019-05-08

**Authors:** Debangana Chakravorty, Krishnendu Banerjee, Tarunendu Mapder, Sudipto Saha

**Affiliations:** 1 Bioinformatics Centre, Bose Institute, Kolkata, India; 2 ARC CoE for Mathematical and Statistical Frontiers, School of Mathematical Sciences, Queensland University of Technology, Brisbane, Australia

**Keywords:** c-Myc, Degradation, Ubiquitination, F-box proteins

## Abstract

**Background:**

c-Myc plays an important role in cell proliferation, cell growth and in differentiation, making it a key regulator for carcinogenesis and pluripotency. Tight control of c-myc turnover is required by ubiquitin-mediated degradation. This is achieved in the system by two F-box proteins Skp2 and FBXW7.

**Results:**

Dynamic modelling technique was used to build two exclusive models for phosphorylation dependent degradation of Myc by FBXW7 (Model 1) and phosphorylation independent degradation by Skp2 (Model 2). Sensitivity analysis performed on these two models revealed that these models were corroborating experimental studies. It was also seen that Model 1 was more robust and perhaps more efficient in degrading c-Myc. These results questioned the existence of the two models in the system and to answer the question a combined model was hypothesised which had a decision making switch. The combined model had both Skp2 and FBXW7 mediated degradation where again the latter played a more important role. This model was able to achieve the lowest levels of ubiquitylated Myc and therefore functioned most efficiently in degradation of Myc.

**Conclusion:**

In this report, c-Myc degradation by two F-box proteins was mathematically evaluated based on the importance of c-Myc turnover. The study was performed in a homeostatic system and therefore, prompts the exploration of c-Myc degradation in cancer state and in pluripotent state.

**Electronic supplementary material:**

The online version of this article (10.1186/s12859-019-2846-x) contains supplementary material, which is available to authorized users.

## Background

c-Myc protein is a short-lived transcription factor that plays an important role in cell proliferation, apoptosis, cell growth, angiogenesis and pluripotency [1]. The structure of c-Myc protein is divided into N- terminal transcription activation domain (TAD) and C-terminal basic-helix-loop-helix-leucine-zipper (bHLH-LZip) domains. The bHLH-LZip region is responsible for dimerization with Max and binding to E-boxes of target gene promoters. Whereas Max is expressed constitutively, Myc is transient. The half-life of c-myc is short (~30mins) in proliferating cells [2] and needs to be tightly regulated as overexpression leads to tumour formation. It is known that c-Myc undergoes ubiquitination followed by degradation in the proteasome [3] and the regions responsible for this signalling is believed to be Myc Box 1 (MBI) and Myc Box 2 (MBII) in the TAD [4]. In fact it is seen that these regions, especially MBI are the hotspots for mutations present in cancer [5].

**Fig. 1:**
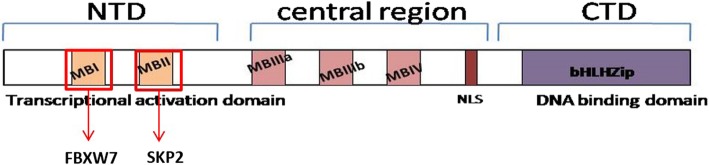
The structure of c-myc with the domain details along with indications of domains that regulate FBXW7 and SKP2, MB-Myc box

Ubiquitin mediated degradation in the proteasomes is enabled by three enzymes; E1 for ubiquitin activation, E2 for ubiquitin conjugation and E3 for ubiquitin ligation which confers substrate specificity [6]. Two types of E3 ubiquitin ligases are there in the system, namely SCF complex and anaphase-promoting complex or cyclosome (APC /C). The SCF complex itself has four components: SKP1, Cul1, Rbx1 and a variable F-box protein which determines target specificity [7]. Among the many F-box proteins identified, Skp2 and FBXW7 have been well characterized in their role of degradation of p27^Kip2^ [8] and Cyclin E [9] respectively. Evidence for binding of both Skp2 and FBXW7 to c-Myc protein is present in literature [10]. Even though both are F-box proteins responsible for degradation, a few key differences may be noted between them. Skp2 contains leucine-rich repeats along with an F-Box domain and binds to c-Myc via MBII and HLH-LZip domains. It degrades c-Myc independent of phosphorylation [11]. FBXW7 on the other hand, contains WD40 repeats along with F-Box and binds to c-Myc via MBI. It requires phosphorylation at S62 and T58 positions for ubiquitination to take place [11] (Fig. 1). Not only do the 2 F-box proteins have different structures and modes of interaction with c-Myc, another important difference lies in the fact that Skp2 is an onco-protein whereas FBXW7 is a tumour suppressor [12].

Although it is known that both FBXW7 and Skp2 regulate c-Myc by degradation, information regarding the distinctions of the tightly regulated degradation process remains to be explored. The turnover of c-Myc plays an important role in the cells decision to proliferate or differentiate in pluripotent cells [13]. Over-expression of Myc is also linked with cancer development [14]. Hence, it is of utmost importance to understand the degradation pathway of c-myc so that any aberrant over-expression can be therapeutically targeted. One of the ways in which control on Myc level is achieved is at the post-translational level via protein stability modulation [15]. Therefore, in this study, role of post-translational modifications in c-Myc function is evaluated giving prime focus on phosphorylation and ubiquitination.

It is already known that sequential phosphorylation of c-Myc takes place by Erk and GSK3β, which is followed by ubiquitination by FBXW7 and degradation in proteasome [16]. Degradation of Myc may also take place independent of the phosphorylation steps by Skp2 protein [17]. Therefore, for our study we have selected these two pathways that degrade c-Myc protein. In addition, questions remain as to how the system chooses which pathway to take for the turnover of Myc. Since experimental studies are time consuming and perturbation of cellular systems require resources, we have aimed to do a preliminary in-silico study to establish the critical steps required for c-Myc degradation. Despite many studies with c-Myc, which resulted in thousands of publication in the past three decades, how Myc expression is still able to get past this regulation and cause cancer is a question that remains unanswered. The results would give us a better insight into the biology of ubiquitination and degradation of c-Myc.

Mathematical modelling has been valuable in assessing the viability of potential therapeutic strategies by identifying critical steps required for regulation. Therefore, to understand the relation between the two types of regulation of c-Myc we have constructed two dynamical models responsible for c-Myc degradation. In the first model, which is a complete and improved version of a model made by Lee et al., we have considered the c-Myc phosphorylation at two residues and then FBXW7 dependent ubiquitination followed by degradation [18]. In the second model, we have dealt with Skp2 mediated ubiquitination of c-Myc independent of phosphorylation. The synchronized consequences of the three signals make the system control the diverse cell fates [19]. We have performed a parameter sensitivity analysis on the two models within the framework of various correlation coefficients to identify the contribution of the modular structures in signal propagation for both the models independently. In the view of signalling and c-Myc degradation, the Fbxw7 mediated pathway shows more robustness over the Skp2 pathway for a large range of alteration in the system components as well as signal components. Questions may arise though as to how Model 1, which is energetically more expensive than Model 2, is the more robust mechanism for c-Myc degradation. To address this query, we have constructed a full model by combining the two independent models in order to see how they perform together. Since it did not fit well with the experimental observations and was not able to efficiently degrade Myc, we incorporated an exclusive ‘on-off switch’ between Model 1 and 2 and tried to find out how and under what circumstances will the system flip from one model to the other.

## Results

### Model building

**Fig. 2:**
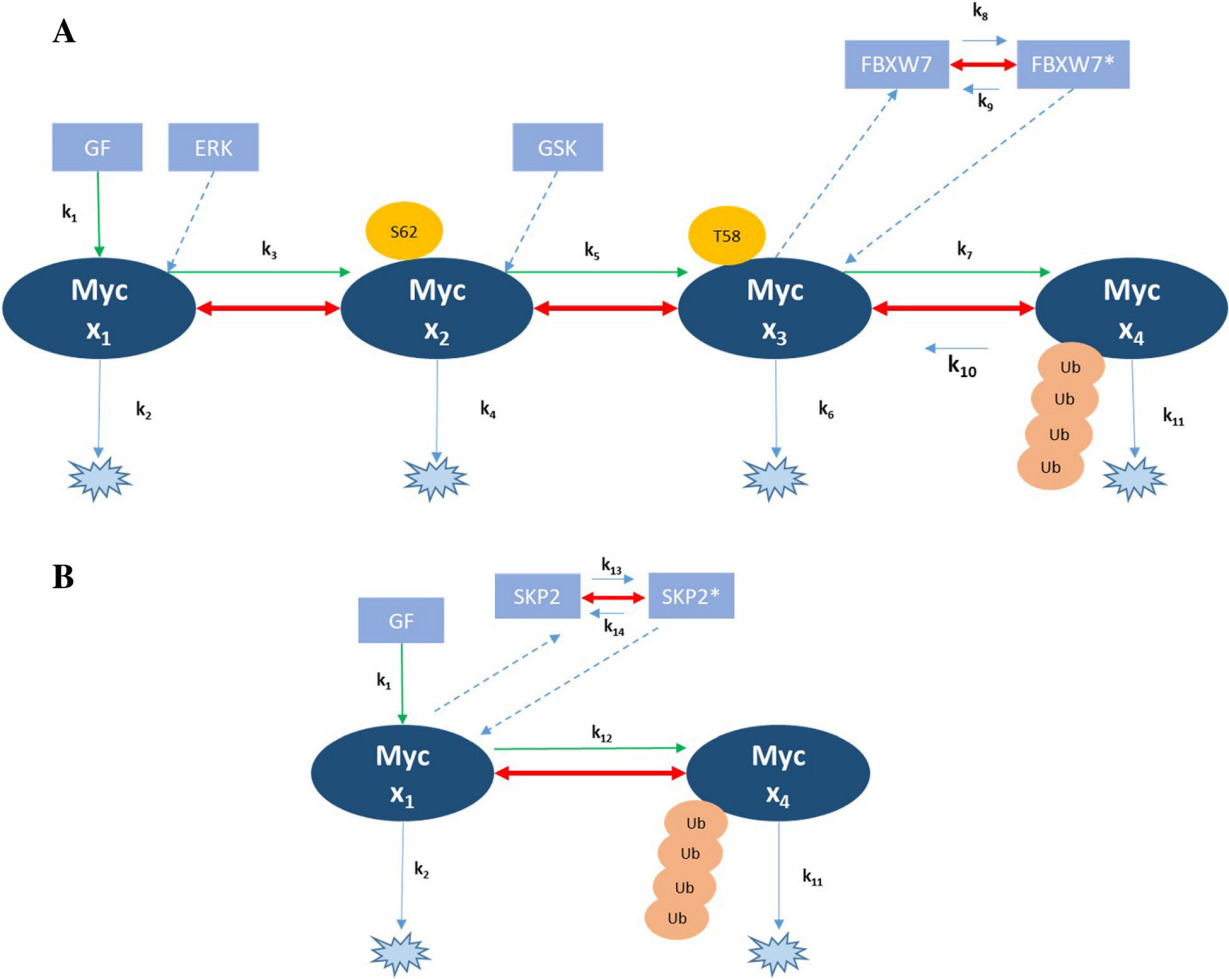
Schematic representation of two models. **a** A schematic representation of Model 1; **b** A schematic representation of Model 2. A: Model 1 (phosphorylation dependent degradation) showing all four states of c-Myc along with the PTMs associated. It also shows the rate constants involved in this model. (GF stands for Growth Factors) B: Model 2 (phosphorylation independent) showing the two states of c-Myc along Skp2. It also shows the rate constants involved in this model. Arrow legends: green lines show conversions between states of Myc, dotted lines show binding reactions, solid lines represent kinetic reactions. For the values and equations of the rate constants, refer to Methods section

In the past few decades, bioinformatics tools have been extensively used in protein degradation related studies. They have also been instrumental in construction of many large scale databases. Nevertheless, these large-scale efforts are mostly restricted in providing only a static picture of a network. We have hypothesized two separate dynamic models (Fig. 2) and explored the possibility of their coexistence for c-Myc degradation taking into consideration normal homeostasis state. This may allow us to predict the changes that may occur in disease state In this study, we have used dynamical model based approach, which is fast evolving into the most promising tool for such purpose.

**Fig. 3:**
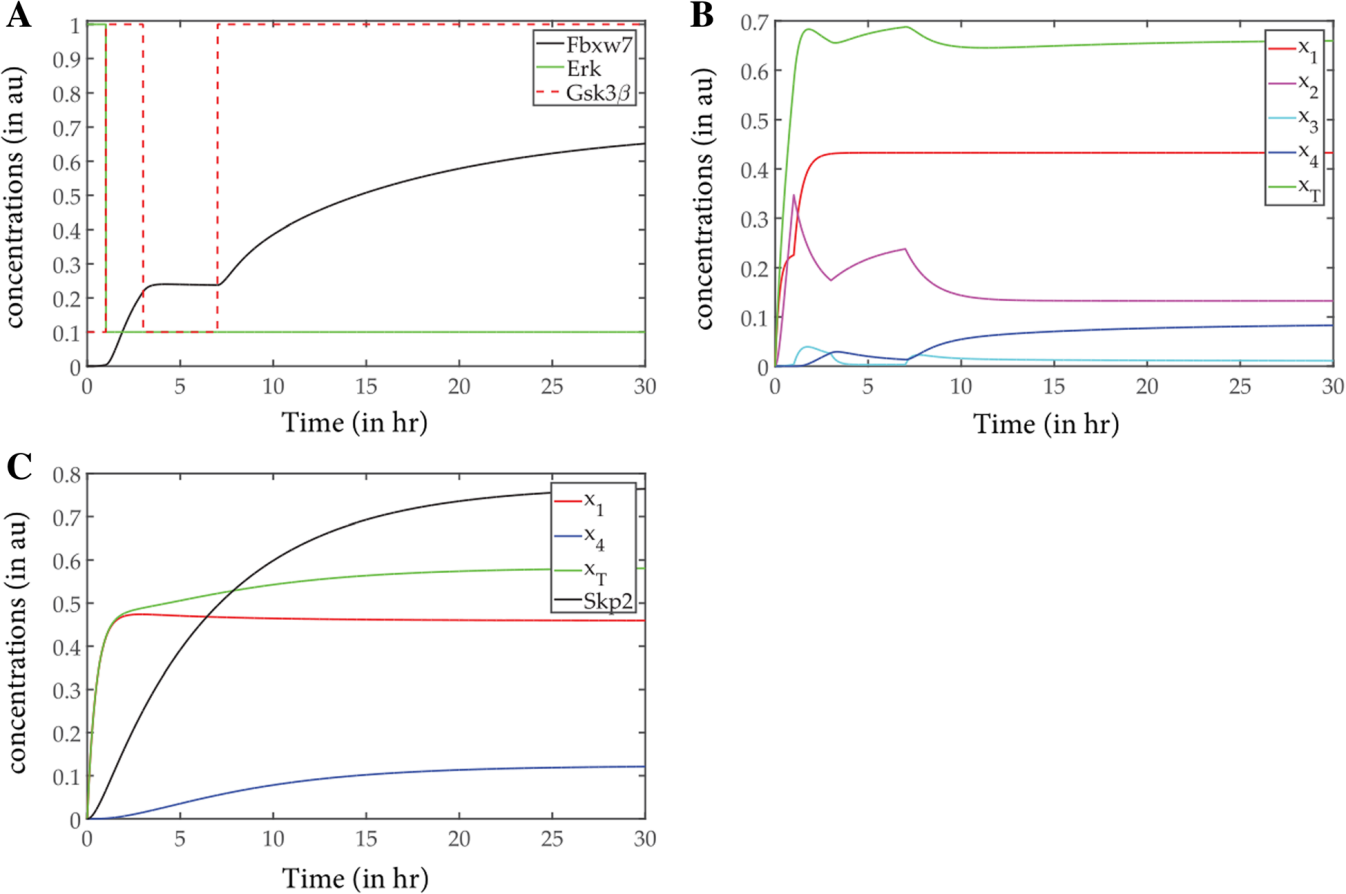
The results obtained from the two models. **a** The signal pulse of of Model 1 with respect to time; **b** The overall population of c-Myc in Model 1 with respect to time; **c** The signal pulse and overall c-myc population of Model 2 with respect to time. **a** The three signals Erk, GSK3β and FBXW7 in the phosphorylation dependent Model 1 with respect to time is given. Erk is denoted in red, Gsk3β in green and FBXW7 in blue. **b** The graph represents levels of c-Myc of the phosphorylation dependent model in all four states (x_1_, x_2_, x_3_ and x_4_) along with total myc concentration (x_T_) with respect to time. The colour coding is given alongside the graph. **c** The signal pulse in phosphorylation independent Model 2, where signal pulse Skp2 is given. In addition, the two states of c-Myc, x_1_ and x_4_ with respect to time is given in the same graph. The colour coding is given alongside the graph

In Model 1, the levels of the signals ERK and GSK were evaluated using the model from Lee et al. and deriving the GSK signal from it [18] (Fig. 3a). As is reported, Myc is highly unstable when synthesized and is stabilized when it undergoes phosphorylation at Serine 62 by Erk [20]. Phosphorylation at Threonine 58 by Gsk3β starts to destabilize Myc and primes it for ubiquitination [21]. It is also seen that in some cell lines Erk has an early transient pulse when stimulated by growth factors (GFs) [22]. On the other hand, PI3K, an antagonist of Gsk3β, has two peaks in some cell lines when stimulated by fetal bovine serum (FBS) [23]. This leads us to derive a signal for Gsk3β having the kind of curve depicted in Fig. 3a [18] (See Methods). It has been experimentally shown that Myc phosphorylations are essential for its binding to Fbxw7 and that Gsk3β inhibitor reduced the interaction between Myc and Fbxw7 [11]. Therefore, we can say that the activation feedback on Fbxw7 comes from x_3_, which is Myc phosphorylated at T58 by GSK, and that Fbxw7 triggers the ubiquitination. The existence of this delayed activation is clearly depicted in the Fig. 3a. Fbxw7 pulse is also generated through assumptions from previous models and experimental results [11] in form of an ODE (Eq) described in the methods section. This part of the Model 1 has been added by our group to the derived version from Lee et al. and extended to phosphorylation dependent ubiquitination models from Nguyen et al. [24]. In this consequence, the on-off timing of the Erk and GSK3β is very crucial. The activation of Myc also integrates and correlates with the input signals. Until Erk stays on (~ 2 h) the pool of c-Myc (x_1–2_) starts growing in a steep fashion, though x_3_ and x_4_ do appear after the Erk signal turns off (Fig. 3b). The most complicated pulsed signal of GSK3β helps the system by introducing delay in the uiquitination process. In presence of these variations in signals, the time profiles of four states of Myc proteins (x_1_–_4_) are generated by solving the set of ODEs (Eq.in Methods) (Fig. 3b).

In the Model 2, the levels of Skp2 signal is regulated in feedback by c-Myc, and encourages the direct ubiquitination process. Experimental evidence suggests that overexpression of Skp2 in Rat1 cells caused enhanced degradation of Myc although in control cells Myc was stabilized [10]. We have derived Model 2 from a generic phosphorylation independent ubiquitination model explained by Nguyen et al [24]. and used experimental data to make approximations of the parameters involved. The kinetics of the two states of c-Myc and Skp2 are evaluated by solving the corresponding dynamical equations (Eq.) and represented in (Fig. 3c). The parameter sensitivity analysis along with the robustness estimation we have performed on the two models gives an idea about the comparative importance of the defined model parameters.

### Sensitivity analysis

**Fig. 4:**
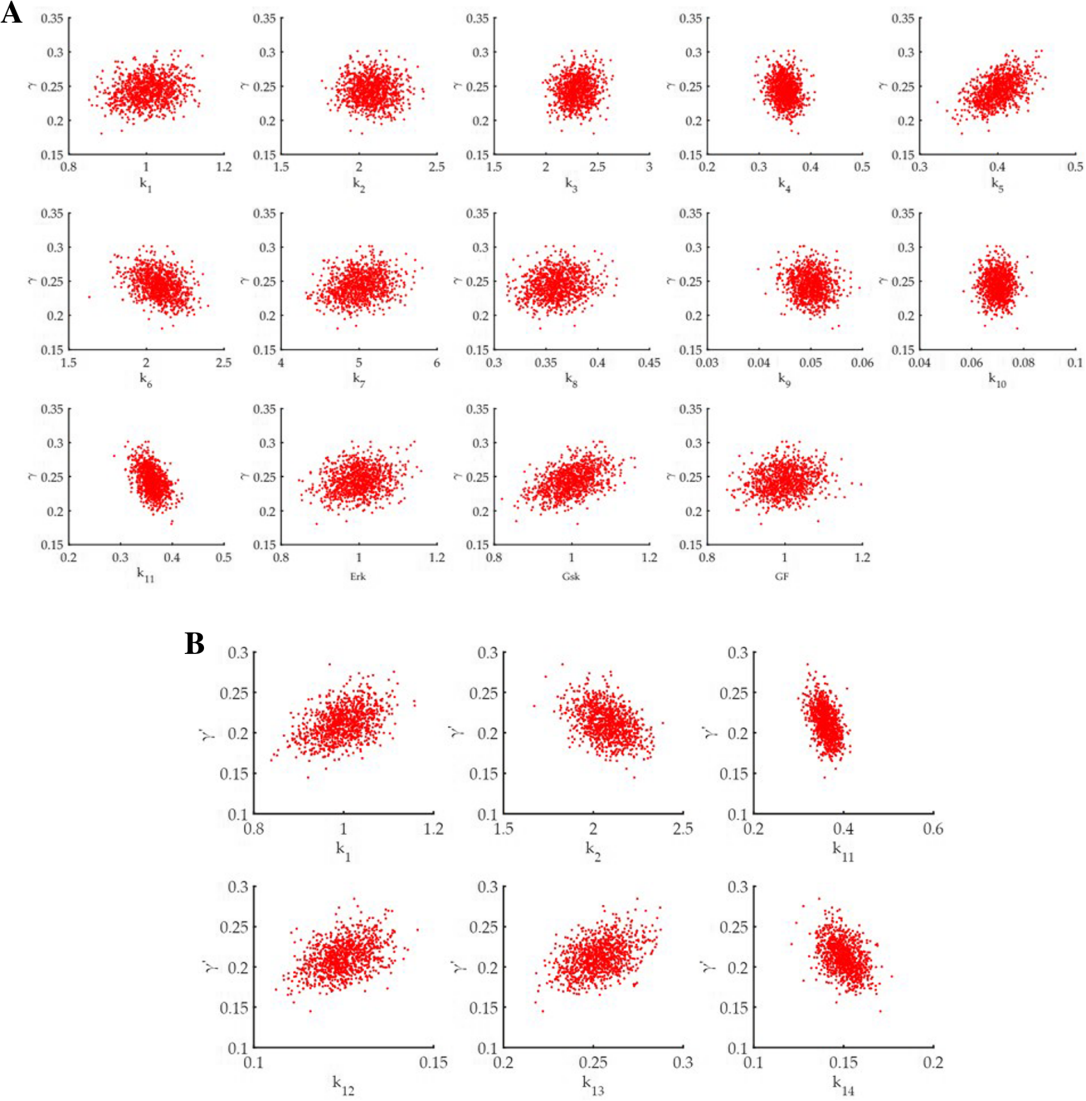
Results of sensitivity analysis. **a** Scatter plot of Model 1; **b** Scatter Plot of Model 2. **a** Scatter plots for the phosphorylation dependent Model 1 for correlation of all parameters against γ. Each parameter is given in the y axis of the scatter plot. **b** Scatter plot for the phosphorylation independent model 2 for correlation of all parameters against γ’. Each parameter is given in the y axis of the scatter plot

For sensitivity analysis, a dimensionless quantity γ (ratio of ubiquitylated Myc with total Myc in all four populations) was calculated for Model 1 and γ’ (ratio of ubiquitylated Myc with total Myc in two populations) for Model 2 (see Methods section). Three types of correlation values were calculated to get an idea of which parameter is most sensitive (Tables 1 and 2). Additionally, scatter plots for the correlation values clearly show the positive and negative correlations (see Methods section for rate constants and other parameters) (Fig. 4a and b). Here, we have computed the correlation indices between the individual model input parameters and the model output. Although we have sampled the model parameters from a normal distribution, it does not make sure that the model output is also normally distributed. In general, for such network of interacting variables, if there exists any nonlinear relation in the model equation, the output variable does not mimic the same distribution as the inputs. If they do, as our results show, then the CC and RCC values will show similar values. In the present study, we are able to check the presence of any nonlinearity in the model by calculating both the CC and RCC. PRCC, on the other hand, can magnify the one-to-one correlation between two variables after removing all the controlling or confounding effects of other model variables. For the case of low strength confounders, the cofactors of each elements would show similar values as the corresponding elements leading to PRCC values same as the RCC values as seen in our results.

**Table 1:** Sensitivity analysis for Model 1 using correlation coefficients

parameters	γ		
	CC	RCC	PRCC
k_1_	0.161503056	0.155807599	0.481319239
k_2_	−0.076018863	− 0.07305514	− 0.248938557
k_3_	0.189660662	0.181862168	0.548371231
k_4_	−0.129866911	−0.123888937	− 0.403739973
k_5_	0.477854476	0.459893896	0.856963902
k_6_	−0.368324896	−0.354892601	− 0.784260863
k_7_	−0.204679	− 0.185476	−0.569011
k_8_	0.156995886	0.148882992	0.481067591
k_9_	−0.149000813	−0.139899758	− 0.457470695
k_10_	−0.040970907	− 0.038100336	−0.135971243
k_11_	−0.462208893	−0.445186864	− 0.848960776
erk	0.188653092	0.181323598	0.55057346
gsk	0.478361213	0.461501541	0.858249977
gf	0.150019829	0.143802131	0.48050302

In the phosphorylation dependent Model 1, the correlation coefficients of γ with all parameters in the model. The table shows all three correlations, CC- Pearson’s correlation coefficient, RCC- Spearman’s rank correlation coefficient and PRCC- partial rank correlation coefficient. k_5_ and gsk have significant positive correlation with γ, whereas k_6_ and k_11_ have significant negative correlation with γ

**Table 2:** Sensitivity analysis for Model 2 using correlation coefficients

parameters	γ’		
	CC	RCC	PRCC
k_1_	0.395439	0.379101	0.672975
k_2_	−0.39888	−0.38966	−0.66149
k_11_	−0.43359	−0.42054	− 0.70365
k_12_	0.583005	0.564045	0.831123
k_12_	0.583079	0.568098	0.831321
k_14_	−0.57593	−0.55415	−1

In the phosphorylation independent Model 2, the correlation coefficients of γ’ with all parameters in the model. The table shows all three correlations, CC- Pearson’s correlation coefficient, RCC- Spearman’s rank correlation coefficient and PRCC- partial rank correlation coefficient. k_12_ and k_13_ have significant positive correlation with γ’, whereas k_14_ has significant negative correlation with γ’

In Model 1, results show that rate constant of GSK3β mediated phosphorylation of x_2_ (k_5_) and GSK3β levels have high positive correlation with γ whereas degradation at Myc T58 phosphorylated x_3_ state (k_6_) as well as degradation of ubiquitylated state x_4_ (k_11_) have negative correlation (Table 1). Experimental evidence from Yada et al. shows that using GSK3 inhibitor reduced the association of Fbxw7 with c-Myc and delayed Myc turnover [11]. This explains the high positive correlation we have found in our model. The correlation coefficients when intensity of perturbation is changed in the range of 5–25% also observes the same trend (data not shown). In Model 2, we see positive correlation of Myc interacting with Skp2 (k_12_) and Skp2 ubiquitylating Myc (k_13_) with γ’, whereas degradation of Skp2 (k_14_) shows a negative correlation (Table 2). This result is reflected in experimental studies by von del Lehr et al. as they show that Skp2 is interacting with Myc in a positive feedback loop where Skp2 deletion mutation led to accumulation of c-Myc [17].

We observe that k_1_ and k_2_ have higher correlation values with γ’ in Model 2 as compared to correlation values with γ in Model 1. It is however noteworthy that degradation of ubiquitylated Myc (k_11_) has higher correlation values (negative correlation) in Model 1 as compared to Model 2. This could indicate that Model 1 is more efficient in degrading Myc. This is also reflected in studies by von der Lehr group which indicates that Skp2 binds with Myc during S phase entry and promotes transcription targets of Myc and Skp2. As they hypothesised that this makes Skp2 mediated degradation a necessary step for activation of transcription [17]. Additionally, Yada et al. have also suggested that inhibition of degradation would be seen only in the initial phase if Skp^−^/^−^ mutation is present in MEFs, as Fbxw7 or other degradation factors will take over the responsibility of degrading Myc later [11]. These evidences may seem to establish Fbxw7 mediated degradation pathway as the more efficient degradation system.

Although t-test was performed for all the correlation coefficients but since the system is linear, performing this analysis did not add any significance to the data (Additional file 1: Table S3 and S4). From these results, we can say that the two models have parity with in-vivo and in-vitro conditions of Myc degradation and correspond to results generated by experimental studies [11, 17].

### Robustness

**Fig. 5:**
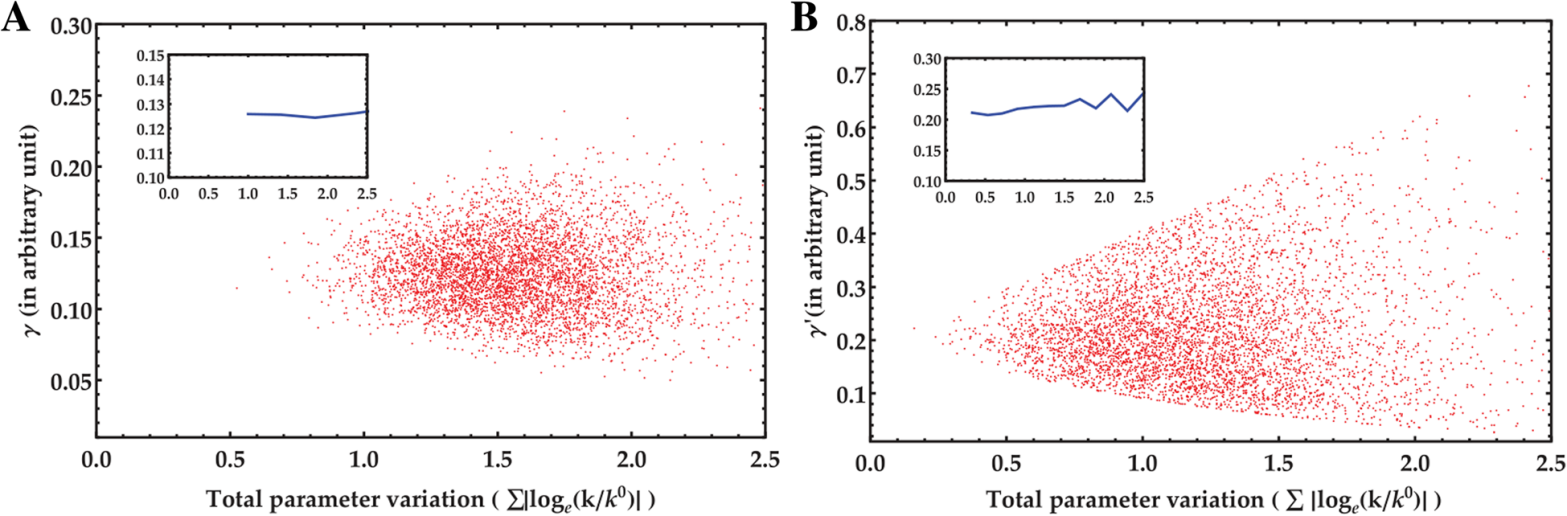
Perturbation analysis. **a** Perturbation analysis for Model 1; **b** Perturbation analysis for Model 2. **a** In the phosphorylation dependent Model 1 the variation in γ is very small with respect to the change in perturbation intensity up to 25%. The figure inset shows the robustness of the model through low variation in moving average of γ with respect to the total parameter variation. **b** In the phosphorylation independent Model 2 the variation in γ’ is very small with respect to the change in perturbation intensity up to 10%. Beyond 10%, some variation was seen. The figure inset shows the robustness of the model through variation in moving average of γ’ with respect to the total parameter variation

In the parameter sensitivity analysis for the two models, we have explored the weight of contribution of each of the model parameters on the model outputs. To check the efficiency of stability of the models, we calculate the robustness in terms of model output with respect to the intensity of perturbation. With the rise of the perturbation intensity on the model parameters, the models generally to lose their stability and deviate far from the stable steady state. From the spread of the scatter plots and the deviations in the moving averages of the model outputs, we can compare the two models quantitatively (Fig. 5a and b). The moving average value calculated for Model 1 stays approximately invariant in the range of the total parameter variation while the same for Model 2 start fluctuating at higher parameter variations. These comparative results simply can infer that Model 1 is more robust than Model 2.

It is known that Skp2 is expressed in S-phase while Fbxw7 is expressed throughout the cell cycle [10]. It is also seen that, overexpression of Fbxw7 reduced transactivation by Myc as compared to overexpression of Skp2, which contributes to growth promoting effects [12]. Finally, we know that mutations in Thr-58 and Ser-62 contributes to cancer [25] therefore giving an importance to these residues for degradation step. From these evidences listed above, we can explain why Model 1 should in fact be more robust than Model 2.

### Decision-making

**Fig. 6:**
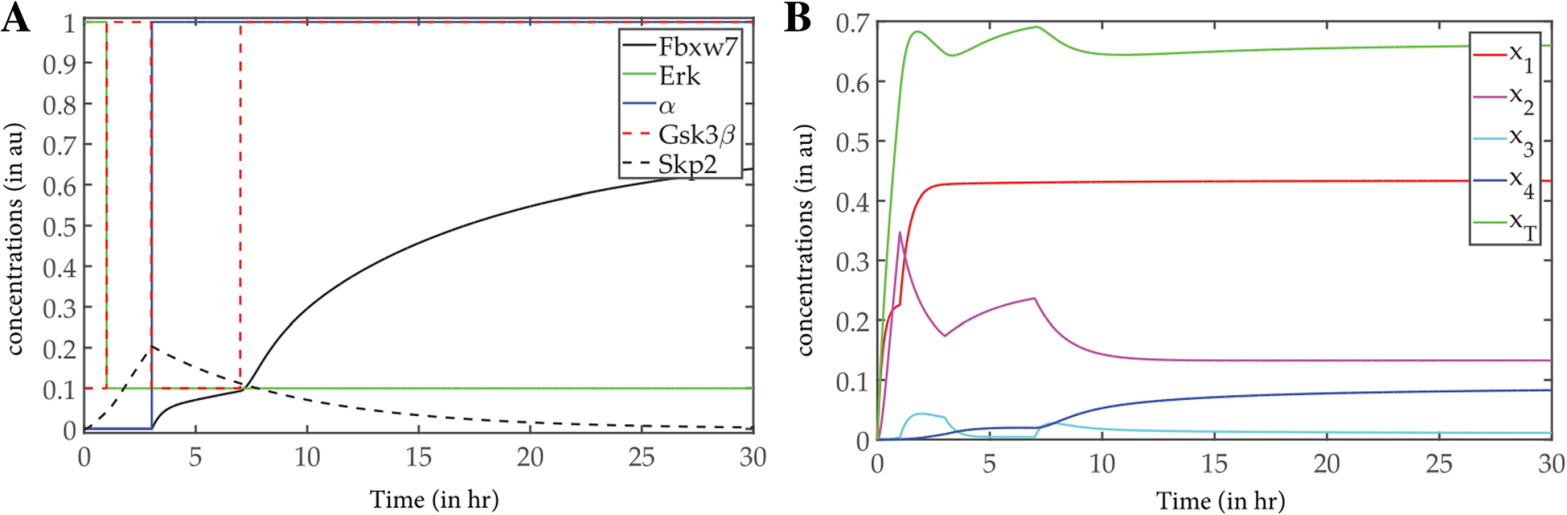
Decision making step of the two models. **a** All input signals and switching function, α with respect to time; **b** The concentration of c-myc populations after incorporating a decision step α. **a** All the input signals along with the switching function α is shown with respect to time. This shows the ‘on’ and ‘off’ states of the decision maker and corresponding activated model inducers. **b** The concentration of c-myc populations after incorporating a decision step α, as to which model will be active and when

In a combined model, if we incorporated every aspect from the two models in logical succession and evaluated the time profiles for the different states of c-Myc it was seen that low concentration of x_4_ could not be reached (Additional file 1: Figure S3). Therefore, based on the information reported till date, it may be concluded that the two models perform exclusively perhaps in different phases of the cell cycle [26, 27]. Infact, as already mentioned before, Skp2 expression is specific to S-phase of the cell cycle. The cell cycle stage or other spatio-temporal factors may therefore lead to the degradation flipping from one pathway to the other. To mimic their exclusive contribution in homeostasis, i.e., the degradation of ubiquitinated c-Myc, we have designed an on-off parameter α that switches between the activation of Fbxw7 and Skp2 (See methods). As model 1 is more dominant in the sense of robustness, we consider it as the on state of α and the model 2 as the off state of α. On sampling the switching function α with different durations and on-off times iteratively, we found two states of α, one ‘on’ states and one ‘off’ state. α stays off up to 3 h from the initial time point and gets on until 30 h, the time course of the observation (Fig. 6a). As an effect of this exclusive activation of the two models, the ubiquitinated c-Myc population is attenuated (Fig. 6b). Our prediction is in line with experimental evidence which suggests that in *Fbw7*^−/−^ ES cells degradation of Myc was impaired for up to 60 min whereas in *Skp2*^−/−^ MEFs Myc degradation was impaired for 20 min [11]. As described before, in homeostasis, it can be concluded that FBXW7 mediated degradation plays the dominant role in degradation of Myc.

## Discussion

The oncoprotein c-Myc, a basic helix-loop-helix/leucine zipper (bHLH/Zip) - type transcription factor, is a master regulator of cell proliferation [28]. The expression of this protein is transient and is responsible for cell proliferation. Its amplification or mutation is present in most types of cancers and therefore, its turnover is a critical determinant of carcinogenesis [29]. Myc is ubiquitinated and marked for degradation in the proteasome [15]. Although we know that regions of Myc like MBI and MBII are not responsible for degradation directly, but they play an important role in binding of E3 ligases [4]. Two such ligases that have been indicated in this paper are FBXW7 and Skp2. Based on experimental evidences and models made by other groups we have discussed two separate models for c-Myc degradation in this paper. Model 1 is a phosphorylation dependent model via FBXW7 whereas Model 2 is a phosphorylation independent model via Skp2.

Dynamic modelling method was used for designing the two models and rate parameters were estimated from experiments as well as models designed by other groups. In this study, for the sake of reducing complexity, we have ignored the reverse reactions in all cases. Other assumptions made include the fact that we have considered the system to be at homeostasis and how the model will function in the diseased state has not been explored. Other limitations include the fact that Skp2 and FBXW7 mediated degradations are not the only two pathways that are responsible for degrading Myc. Other pathways have been ignored for making the model simple.

From the results obtained in our models and comparing them with experimental data cited we can conclude that the mechanisms of degradation hypothesised by us is in line with experimentally proved in the biological system. The results also indicate that Model 1 is more robust and degradation rate constant k_11_ has a higher correlation with output signal γ. This happens because efficient degradation of c-Myc requires a protein, which is a tumour suppressor, like FBXW7. Skp2 on the other hand is an oncoprotein, which along with ubiquitylating Myc also helps in its transactivation. The role of Skp2 remains unclear, but it is evident that Model 1 is a more reliable method of regulating c-Myc. This is also evident as mutations in S62 and T58, residues involved in phosphorylation according to model 1, are responsible for cancer phenotype [25].

To find out why two different mechanisms exist in vivo and to understand its role on degradation of Myc, we have combined the two models. Results indicate that when these two models work together simultaneously, they are not efficient to reduce the ubiquitylated Myc population (Additional file 1: Figure S3). This may imply that even in the biological system, the two methods for degradation work exclusively. Therefore, a switch was incorporated to decide which E3 ligase would work at which point of time. In the cell, it is perhaps the cell cycle state, which decides when the two E3 ligases operate. It can be concluded that by combining the two models in a decision dependent manner lower levels of ubiquitylated Myc could be achieved. It should also be noted that in the combined model, FBXW7 plays a more predominant role than Skp2 in c-Myc degradation.

We can find some clues from this combined model as to which steps are critical in causing Myc over-expression leading to cancer. However, the entire dynamic modelling was done for the system in homeostasis. Myc is known to play an important role in cancer state as well as in pluripotency. It is an important player, which modulates expression of multiple other genes to switch from normal state to cancer or from non-dividing state to proliferative state [30]. This area remains to be explored further and the use of model-based methods to decipher therapeutic strategies remains to be the target for researchers. It is also expected that modelling and model-based approaches in integration with experimentation will become a major tool for data interpretation and hypothesis generation in all fields of biology.

## Conclusion

c-Myc is a transcription factor responsible for cell fate decisions. The presence of many F-box proteins functioning as E3 ubiquitin ligase for degradation of c-Myc protein has been reported. In this study two such proteins Skp2 and FBXW7 have been explored for their role in degradation. It has been shown that phosphorylation dependent degradation via FBXW7 is a more robust mechanism for degradation in spite of which phosphorylation independent degradation via Skp2 also takes place. Therefore a putative mechanism of degradation that takes place in the system has been hypothesised by combining the two models in a decision dependent manner. This gives way to understanding how c-Myc may be regulated in the cell and question how in disease-state as well as during pluripotency this process is altered.

## Methods

Phosphorylation and ubiquitination often have opposing effects on target proteins and require the interaction of different partners. This creates complications and makes the model quite large, which renders model construction and parameter estimation quite challenging. In this study, not only have we combined phosphorylation with ubiquitination, but we have also given two alternative pathways by which Myc can be ubiquitinated. Finally, we have combined the two models to give a better idea as to what happens in the system.

### Phosphorylation dependent c-Myc degradation: model 1

The role of post-translational modifications (PTMs) in c-Myc was investigated in a kinetic modeling framework. Myc is stimulated by external growth factors (*GF*) and further gets a sequential phosphorylation by Erk (*E*) and GSK3β (*G*) at Serine 62 (S62) and Threonine 58 (T58) respectively. The T58 phosphorylation occurs with the dephosphorylation at S62. The T58 phosphorylation leads to activation of the F-box protein FBXW7, which eventually triggers the ubiquitination of Myc and hence degradation (Fig. 2). To make the model simplified and tractable, we have not considered the phosphatases and the DUBs in the present network. The four states of c-Myc are depicted by *x*_*1*_, *x*_*2*_, *x*_*3*_, and *x*_*4*_ for *GF* stimulated c-Myc, c-Myc phosphorylated at S62, c-Myc phosphorylated at T58 and ubiquitinated, respectively. The kinetic reactions of the cascade have been depicted in Additional file 1: Table S1 along with reaction rates. The rate constants were derived from previous models and were subjected to estimation and assumptions [18, 24, 31, 32].

The Erk and GSK3β signals were introduced in pulses of different structures, while the GF induces the c-Myc activation constantly. The Pulse amplitudes were kept at 1.0 but the on-off switch durations for Erk and GSK3β are different. Erk was on up to 1.0 h and got turned off as soon as GSK3β signal turned on. The nature of the GSK3β signal was complicated; it turned on at 1.0 h, was turned off at 2.0 h and again turned on at 7.0 h and stayed on until the end of the time course. All rate parameters can be found in the Additional file 1: Table S1.The previously mentioned degradation cascade can be presented in a set of coupled ordinary differential equations as

$$ {\displaystyle \begin{array}{l}\frac{dx_1}{dt}={k}_1 GF-{k}_2{x}_1-{k}_3{Ex}_1\\ {}\frac{dx_2}{dt}={k}_3{Ex}_1-{k}_4{x}_2-{k}_5{Gx}_2\\ {}\frac{dx_3}{dt}={k}_5{Gx}_2-{k}_6{x}_3-{k}_7F\ast {x}_3+{k}_{10}{x}_4\\ {}\frac{dx_4}{dt}={k}_7F\ast {x}_3-\left({k}_{10}+{k}_{11}\right){x}_4\\ {}\frac{dF\ast }{dt}={k}_8{x}_3\left({F}_T-F\ast \right)-{k}_9F\ast \end{array}} $$

Where, the pulse of Erk and GSK3β are constructed by the combination of several Heaviside step functions as

$$ {\displaystyle \begin{array}{l}E={E}_R+{E}_{Max}\theta \left({Dur}_E-t\right)\\ {}G={G}_R+{G}_{Max}\left(\pi \left[{Width}_G\left({Dur}_{G1}-t\right)\right]+\theta \left(t-{Dur}_{G2}\right)\right)\end{array}} $$

The system of ODEs has been solved for 30 h, as described in a previous model [18]. A dimensionless fraction of ubiquitinated c-Myc (x_4_/(x_1_ + x_2_ + x_3_ + x_4_) = γ) has been considered as the model output. At steady state, we performed an input parameter sensitivity analysis and checked the robustness of the model with respect to this model output. The methodology of the correlation coefficient based sensitivity analysis and robustness calculation will be described later.

Concentration of growth factor: *GF* = 1.0;

Details of the Erk pulse: *E*_*Max*_ = 0.9; *E*_*R*_ = 0.1; *Dur*_*E*_ = 1.0;

Details of the Gsk pulse: *G*_*Max*_ = 0.9; G_R_ = 0.1; *Dur*_*G1*_ = 2; *Dur*_*G2*_ = 7; *Width*_*G*_ = 0.5;

Details of the Fbxw7 pulse: *F*_*T*_ = 5.0.

### Phosphorylation independent c-Myc degradation: model 2

Apart from the Fbxw7 dependent pathway of c-Myc degradation, a Skp2 mediated pathway exists and it is independent of phosphorylation. The Skp2 pathway of c-Myc degradation is triggered by the activation of Skp2 by c-Myc. Skp2 starts ubiquitination of c-Myc for degradation in the proteasome. We have framed the model in a set of ordinary differential equations as

$$ {\displaystyle \begin{array}{l}\frac{dx_1}{dt}={k}_1 GF-{k}_2{x}_1-{k}_{12}{x}_1S\\ {}\frac{dx_4}{dt}={k}_{12}{x}_1S-{k}_{11}{x}_4\\ {}\frac{dS}{dt}={k}_{13}{x}_1-{k}_{14}S\end{array}} $$

where, *x*_*1*_, *x*_*4*_ are the states of c-Myc before and after ubiquitination and *S* stands for Skp2. The detail of the reactions is tabulated in Additional file 1: Table S2. Similarly, a dimensionless fraction of ubiquitinated c-Myc (x_4_/(x_1_ + x_4_) = γ’) has been considered as the model output. Some parameters were taken from the previous model.

### Parameter sensitivity

To quantify the contribution of each model parameter simultaneously, we prefer to utilize the correlation coefficient based parameter sensitivity analysis. We calculate three kinds of correlations, the Pearson’s (CC), Spearman’s rank (RCC), and the partial rank (PRCC) correlation coefficients as reported in a previous article [33]. All the correlation coefficients were measured at steady state only with increasing the strength of perturbation, but it does not show any notable change until 20%. Within the period of 30 h, the Erk signal goes ‘off’ from ‘on’ state once and the Gsk signal flips thrice. We try to find the variation in the parameter sensitivity profiles near these flip points for Model 1, but we cannot observe any difference. p –values was calculated for both models using t-test and results were tabulated in Additional file 1: Tables S3 and S4.

### Robustness

The efficiency of a biochemical network to maintain its functionality in the very altering environment can be quantitated. Measure of the model robustness is one of the simple and reliable approaches. In the current paper, robustness of the two models are analysed in terms of ensembles of perturbed systems. The rate parameters and system inputs like the duration and width of the Erk and Gsk signals were sampled randomly from Gaussian distribution about their native values and corresponding spread of the network output (γ and γ’) is measured. The robustness was characterized in terms of the summation of normalized alteration in the parameter set for an ensemble of 5000 models [34]. We increase the perturbation intensity and note the ensemble of the variation in the γ and γ ‘-value from the original.

### Decision process

The previously mentioned two models of c-Myc degradation exist in the system but perform exclusively. We predict there must be some hidden agents, who decide the switch between the two models. To monitor the decision making process, we assemble the two models in to a single one and consider the activation of Fbxw7 and Skp2 as exclusive events.

$$ {\displaystyle \begin{array}{l}\frac{dx_1}{dt}={k}_1 GF-{k}_2{x}_1-{k}_3{Ex}_1-{k}_{12}{x}_1S\\ {}\frac{dx_2}{dt}={k}_3{Ex}_1-{k}_4{x}_2-{k}_5{Gx}_2\\ {}\frac{dx_3}{dt}={k}_5{Gx}_2-{k}_6{x}_3-{k}_7F\ast {x}_3+{k}_{10}{x}_4\\ {}\frac{dx_4}{dt}={k}_7F\ast {x}_3-\left({k}_{10}+{k}_{11}\right){x}_4+{k}_{12}{x}_1S\\ {}\frac{dF\ast }{dt}=\alpha {k}_8{x}_3\left({F}_T-F\ast \right)-{k}_9F\ast \\ {}\frac{dS}{dt}=\left(1-\alpha \right){k}_{13}{x}_1-{k}_{14}S\end{array}} $$

where α is the switching parameter, flips between 0 and 1. When Fbxw7 is activated, the model 1 will be on board whether activation of Skp2 triggers the model 2 in action. To explore the switching time and preference of the two models we have defined a switching parameter α that triggers activation of the models exclusively at preferred time points. The combined model was framed as an optimization problem with an objective to reduce the population of x_4_ in the system over the time course. To perform the optimization, we have constructed an objective function as

$$ J={\int}_0^{30}{x}_4(t) dt $$

to be minimized. To optimize the temporal evolution of α throughout the course of the full signaling network, we need to construct a generalized step switch with flexible ‘on-off’ at any time. A generalized Pi function has been considered with duration and ‘on-off’ time sampled from a uniform distribution over the full timescale. On running 10,000 independent iterations, we have found the optimal structure of the switching function. For the optimization runs and series of sampled structures of the switch please find the Additional file 1: Figure S1-S2.

## Additional files

Additional file 1: **Figure S1.** Few representative replicas from the sample set of the switching function gives better understanding of the different switching time and states. **Figure S2.** The profile of the objective function (*J*) over 100 samples. We have minimized *J* over 10,000 independent sample runs. **Figure S3.** Combined Model of Model 1 and Model 2. A: All input signals of the two models are combined and shown vs time; B: The concentration of c-myc populations with respect to time is shown. Colour codes are given for both graphs. Additionally we can observe that × 4 value is not as low as expected (Compare with Fig. 6). **Table S1.** Reaction scheme of the phosphorylation dependent degradation of c-Myc, Model 1. **Table S2.** Reaction scheme of the phosphorylation independent degradation of c-Myc, Model 2. **Table S3.** *p*-values for the correlation coefficients in Model 1. **Table S4.** p-values for the correlation coefficients in Model 2. (DOCX 560 kb) (DOCX 560 kb)

Additional file 2: This file contains software specific codes for all the analysis performed in this study including the codes for the two combined models in a pfd format. (RAR 10429 kb)

## Abbreviations

APC /C: Anaphase-promoting complex or cyclosome
bHLH-LZip: Basic-helix-loop-helix-leucine-zipper
CC: Pearson’s correlation coefficient
Erk: Extracellular signal–regulated kinases
ES cells: Embryonic stem cells
FBS: Fetal bovine serum
FBXW7: F-Box And WD Repeat Domain Containing 7
GF: Growth factors
GSK3β: Glycogen synthase kinase-3 beta
MBI: Myc Box 1
MBII: Myc Box 2
MEFs: Mouse embryonic fibroblasts
ODEs: Ordinary differential equations
PI3K: Phosphatidylinositol-3-kinases
PRCC: Partial rank correlation coefficient
Rbx1: RING-box protein 1
RCC: Spearman’s rank correlation coefficient
S62: Serine at position 62
SCF: Skp, Cullin, F-box containing complex
Skp1: S-phase kinase-associated protein 1
Skp2: S-phase kinase-associated protein 2
T58: Threonine at position 58
TAD: Transcription activation domain
