# Supplementary material for: In silico modeling of phosphorylation dependent and independent c-Myc degradation
Source: BMC Bioinformatics. 2019 May 8;20:230. doi: 10.1186/s12859-019-2846-x (PMC6505206; doi:10.1186/s12859-019-2846-x)
Supplement: Supplementary file 1 — Figure S1. Few representative replicas from the sample set of the switching function gives better understanding of the different switching time and states. Figure S2. The profile of the objective function (J) over 100 samples. We have minimized J over 10,000 independent sample runs. Figure S3. Combined Model of Model 1 and Model 2. A: All input signals of the two models are combined and shown vs time; B: The concentration of c-myc populations with respect to time is shown. Colour codes are given for both graphs. Additionally we can observe that × 4 value is not as low as expected (Compare with Fig. 6). Table S1. Reaction scheme of the phosphorylation dependent degradation of c-Myc, Model 1. Table S2. Reaction scheme of the phosphorylation independent degradation of c-Myc, Model 2. Table S3. p-values for the correlation coefficients in Model 1. Table S4. p-values for the correlation coefficients in Model 2. (DOCX 560 kb) (DOCX 560 kb) [file 12859_2019_2846_MOESM1_ESM.docx]

***In silico* modeling of phosphorylation dependent and independent c-Myc degradation**

**Debangana Chakravorty^1^, Krishnendu Banerjee^1^, Tarunendu Mapder^2*^, Sudipto Saha^1*^**

**1. Bioinformatics Centre, Bose Institute, Kolkata, India**

**2. ARC CoE for Mathematical and Statistical Frontiers, School of Mathematical Sciences, Queensland University of Technology, Brisbane, Australia**

**Supplementary information**

**Supplementary Figure S1:** Few representative replicas from the sample set of the switching function gives better understanding of the different switching time and states.

**Supplementary Figure S2:** The profile of the objective function (*J*) over 100 samples. We have minimized*J*over 10000 independent sample runs.

**Supplementary Figure S3:** Combined Model of Model 1 and Model 2. **A:** All input signals of the two models are combined and shown vs time; **B:** The graph represents levels of c-Myc of the combined model in all four states (x_1_, x_2_, x_3_ and x_4_) along with total Myc concentration (x_T_) with respect to time. Colour codes are given for both graphs. Additionally we can observe that x4 value is not as low as expected (Compare with Figure 6).

**Supplementary Table S1:** Reaction scheme of the phosphorylation dependent degradation of c-Myc, Model 1

**Supplementary Table S2:** Reaction scheme of the phosphorylation independent degradation of c-Myc, Model 2

**Supplementary Table S3:** p-values for the correlation coefficients in Model 1.

**Supplementary Table S4:** p-values for the correlation coefficients in Model 2.


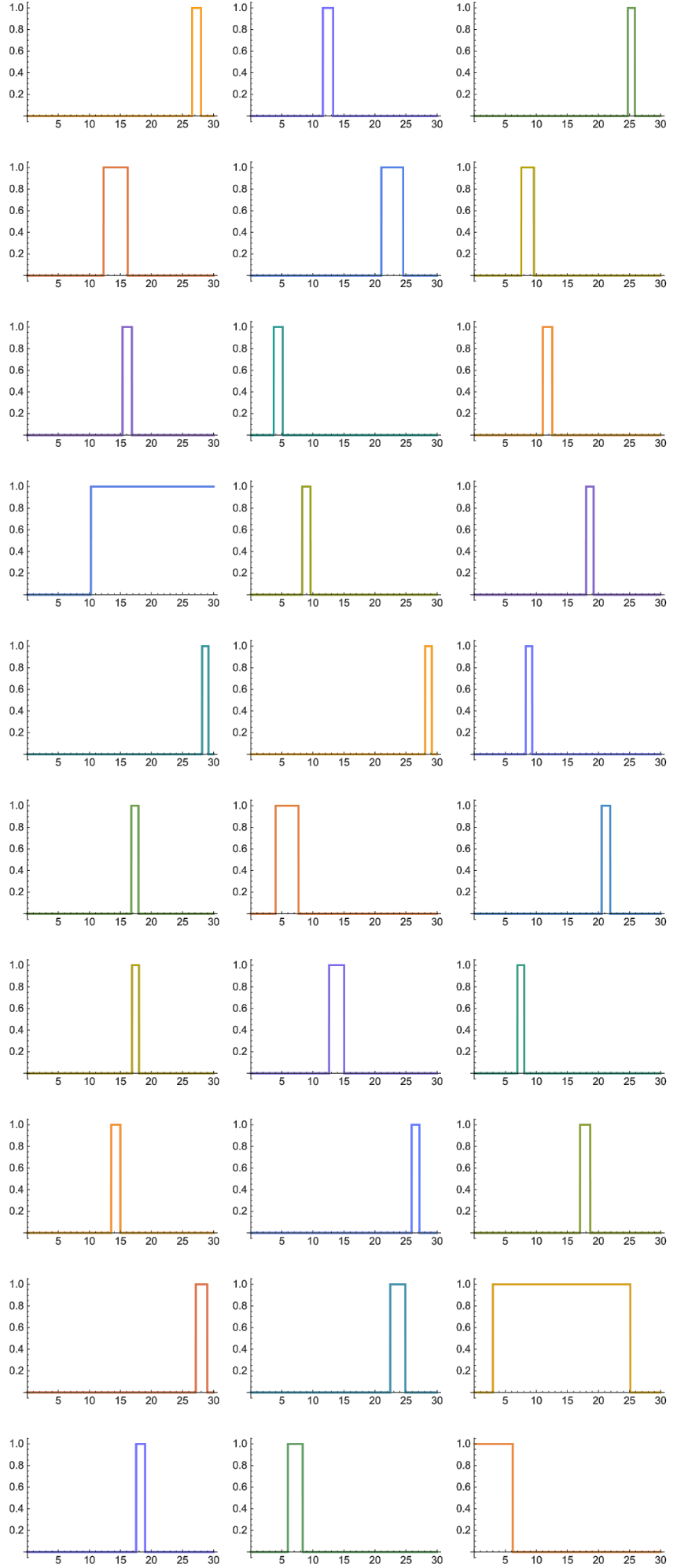


**Supplementary Figure S1**

**
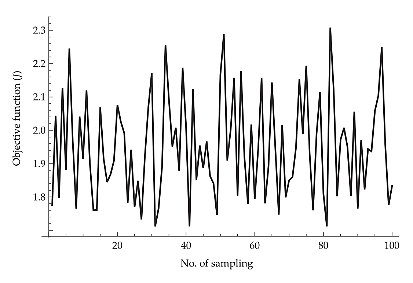
**

**Supplementary Figure S2**


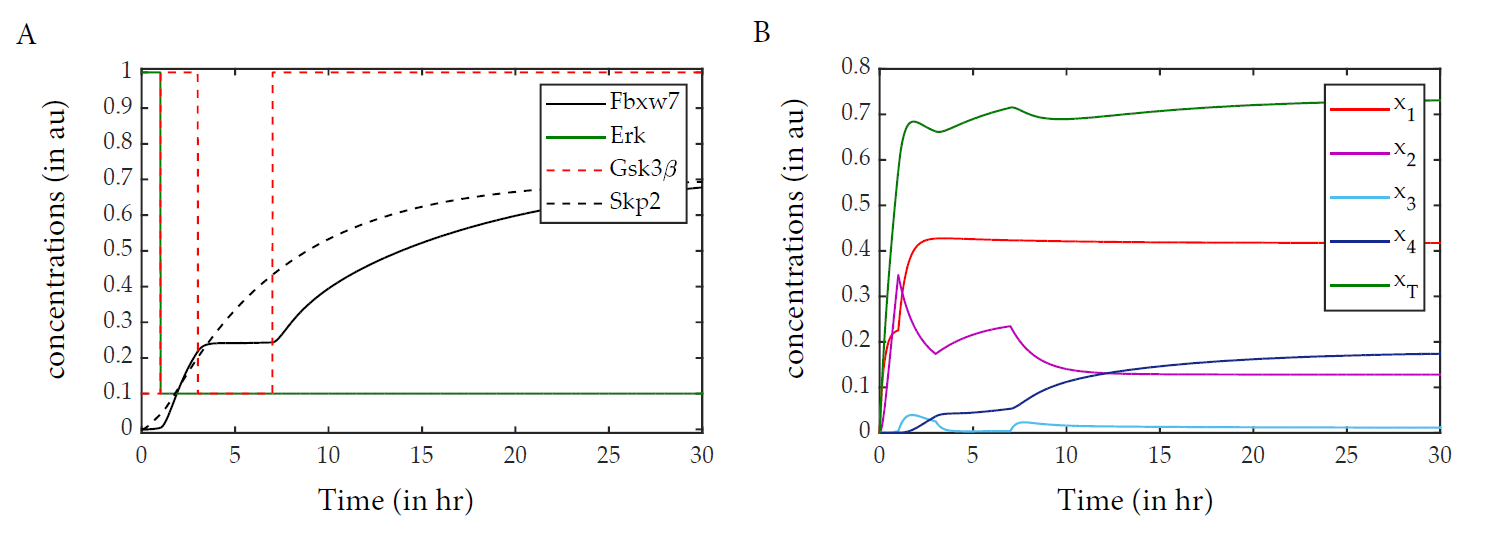


**Supplementary Figure S3**

**Supplementary Table S1**

| Description | Reaction | Parameter |
| --- | --- | --- |
| Growth factor mediated activation of c-Myc | (GF)🡪 x_1_ | k_1_= 1.0 molecules-hr^-1^ |
| Degradation of c-Myc | x_1_🡪ø | k_2_= 2.8 hr^-1^ |
| Erk mediated phosphorylation of x_1_ | x_1_+E🡪 x_2_ | k_3_= 2.3 molecules_­_^-1^ -hr^-1^ |
| Degradation of x_2_ | x_2_🡪ø | k_4_=0.35 hr^-1^ |
| GSK3β mediated phosphorylation of x_2_ | x_2_+G🡪x_3_ | k_5_= 0.4 molecules_­_^-1^ -hr^-1^ |
| Degradation of x_3_ | x_3_🡪ø | k_6_= 2.08 hr^-1^ |
| FBXW7 mediated ubiqutination of x_3_ | x_3_+F*🡪x_4_ | k_7_= 5.0 molecules_­_^-1^ -hr^-1^ |
| Activation of FBXW7 by x_3_ | F+x_3_🡪F* | k_8_= 0.36 molecules_­_^-1^ -hr^-1^ |
| Deactivation of FBXW7 | F*🡪F | k_9_= 0.05 hr^-1^ |
| Removal of ubiquitin from x_4_ | x_4_🡪x_3_ | k_10_= 0.07 hr^-1^ |
| Degradation of x_4_ | x_4_🡪ø | k_11_= 0.36 hr^-1^ |

**Supplementary Table S2**

| Description | Reaction | Rate parameter |
| --- | --- | --- |
| Growth factor mediated activation of *x_1_* | (GF) 🡪*x_1_* | k_1_= 1.0 molecules-hr^-1^ |
| Degradaition of x_1_ | x_1_🡪ø | k_2­_= 2.08 hr^-1^ |
| Skp2 mediated ubiquitination of x_1_ | x_1_+S*🡪x_4_ | k_12_= 0.125 molecules^-1^-hr^-1^ |
| Activation of Skp2 by x_1_ | x_1_+ S🡪S* | k_13_= 0.252 molecules-hr^-1^ |
| Degradation of x_4_ | x_4_🡪ø | k_11_= 0.36 hr^-1^ |
| Deactivation of Skp2 | S*🡪S | k_14_= 0.15 hr^-1^ |

**Supplementary Table S3**

| parameters | γ | | |
| --- | --- | --- | --- |
|  | CC | RCC | PRCC |
| k_1_ | 4.48e-52 | 7.04E-49 | 0 |
| k_2_ | 5.92E-11 | 5.58E-10 | 6.05E-115 |
| k_3_ | 2.35E-70 | 1.94E-63 | 0 |
| k_4_ | 4.64E-33 | 2.60E-31 | 0 |
| k_5_ | 0 | 0 | 0 |
| k_6_ | 0 | 0 | 0 |
| k_7_ | 3.28E-128 | 0 | 0 |
| k_8_ | 1.33E-56 | 6.81E-55 | 0 |
| k_9_ | 2.19E-52 | 9.34E-50 | 0 |
| k_10_ | 1.24E-4 | 6.95E-4 | 1.65E-38 |
| k_11_ | 0 | 0 | 0 |
| erk | 2.16E-81 | 0 | 0 |
| gsk | 0 | 0 | 0 |
| gf | 7.92E-57 | 1.10E-51 | 0 |

**Supplementary Table S4**

| parameters | γ' | | |
| --- | --- | --- | --- |
|  | CC | RCC | PRCC |
| k_1_ | 0 | 0 | 0 |
| k_2_ | 0 | 0 | 0 |
| k_11_ | 0 | 0 | 0 |
| k_12_ | 0 | 0 | 0 |
| k_12_ | 0 | 0 | 0 |
| k_14_ | 0 | 5.67E-315 | 0 |
